# Genetically Triggered Thoracic Aortic Disease: Who Should be Tested?

**DOI:** 10.14797/mdcvj.1218

**Published:** 2023-03-07

**Authors:** Valeria E. Duarte, Raman Yousefzai, Michael N. Singh

**Affiliations:** 1 Houston Methodist DeBakey Heart & Vascular Center, Houston, Texas, US; 2 Boston Children’s Hospital, Boston, Massachusetts, US; 3 A.T. Still University-Kirksville College of Medicine (ATSU-KCOM), Kirksville, Missouri, US; 4 Boston Children’s Hospital and Harvard Medical School, Boston, Massachusetts, US; 5 Brigham and Women’s Hospital, Harvard Medical School, Boston, MA, US

**Keywords:** heritable thoracic aortic diseases (HTAD), thoracic aortic aneurysms (TAD), Marfan syndrome (MFS), genetic testing, nonsyndromic HTAD, genetic variants

## Abstract

Up to 25% of patients with thoracic aortic disease have an underlying Mendelian pathogenic variant. This is a heterogeneous group of disorders known as heritable thoracic aortic diseases (HTAD). Diagnosing associated pathogenic gene variants and syndromes is critical, as the underlying genetics have an implication in medical management, surveillance, thresholds for surgical intervention, surgical risk, pregnancy risk, and risk of inheritance by the offspring. Recently released 2022 American College of Cardiology/American Heart Association guidelines for the diagnosis and management of aortic diseases provide specific recommendations to identify patients at risk for heritable conditions and who should undergo genetic testing.

## Introduction

In one population study, the incidence of thoracic aortic aneurysms was 10.4 per 100,000 person-years, showing a three-fold increase in the rate in 1980 to 1994 compared with 1951. In addition, 51% of the patients with thoracic aortic aneurysm were identified in women, who were considerably older at recognition than men.^1^ Most patients with thoracic aneurysms are asymptomatic and diagnosed incidentally during chest or cardiac imaging or after they present as life-threatening emergencies with dissection or rupture. Unfortunately, in a significant number of patients, the diagnosis is made after death.

The recently released 2022 American College of Cardiology (ACC)/American Heart Association (AHA) guidelines for the diagnosis and management of aortic disease has specific recommendations for identifying patients at risk for heritable conditions who should undergo genetic testing. For risk assessment, it is critical to obtain a multigenerational family history of thoracic aortic disease (TAD), sudden cardiac deaths, and peripheral and intracranial aneurysms.^2^

## Genetic Etiology of Thoracic Aortic Disease

Up to 20% to 25% of patients with TAD have an underlying Mendelian pathogenic variant (Table 1). These conditions are known as heritable thoracic aortic diseases (HTAD), a clinically and genetically heterogeneous group of disorders. Identifying pathogenic genes is essential to identify family members at risk for the disease, guide surveillance and management, and evaluate associated vascular and systemic complications.^2,3^

**Table 1:** Pathogenic variants associated with heritable thoracic aortic disease (HTAD).


TYPE OF HTAD	GENETIC ABNORMALITY

**Syndromic HTAD**	

Marfan Syndrome	*FBN1*
Loeys-Dietz Syndrome	*TGFBR1 TGFBR2 SMAD2* *SMAD3* *TGFB2*, *TGFB3*
Vascular Ehlers-Danlos Syndrome (type IV)	*COL3A1*

**Nonsyndromic HTAD**	*ACTA2, MYH11, MYLK, LOX*, and *PRKG1*


From a pathological perspective, patients with HTAD show destructive matrix remodeling with elastin fragmentation, proliferation of vascular smooth muscle cells, and a less prominent inflammatory component without atheroma. Eleven genes are confirmed to cause a highly penetrant risk for HTAD: *FBN1, LOX, COL3A1, TGFBR1M, TGFBR2, SMAD3, TGFB2, ACTA2, MYH11, MYLK*, and *PRKG1*.^2,3^ Moreover, HTAD can be subdivided into syndromic and nonsyndromic.^4^ Syndromic HTAD is associated with genetic syndromes with multisystem involvement, the most common being Marfan syndrome (MFS), followed by other less frequent connective tissue syndromes including Loeys-Dietz (LDS) and vascular Ehlers-Danlos (EDS).

The other group, nonsyndromic, comprises a group of patients with an underlying genetic pathogenic variant (mutation) that affects the aorta and, in some, its branches, but no other systems. In addition, there is a group of patients with a family history of TAD with genetic mutation that have unknown pathologic significance or no mutation has been identified. Notably, among the patients with TAD without a diagnosis of MFS or LDS, up to 20% have a first-degree relative with similar features.^5,6^ Thoracic aortic aneurysms also are associated with Turner syndrome, bicuspid aortic valve, coarctation of the aorta, and other congenital conditions that exceed the scope of this document.

## Aortic Disease Associated with Genetic Syndromes

Marfan syndrome, Loeys-Dietz syndrome, and vascular Ehlers-Danlos syndrome are the most recognized genetic syndromes associated with HTAD.

### Marfan Syndrome

Marfan syndrome is a systemic disorder of the connective tissue. It is caused by the mutation in the *FBN1* gene inherited in an autosomal dominant fashion. The *FBN1* gene encodes brillin-1, a glycoprotein that is a major structural component of the extracellular matrix that supports the connective tissues.^7,8^ The prevalence of MFS ranges between 1 in 5,000 and 1 in 10 000, without any ethnic or gender bias.^9^ MFS has high penetrance and variable expression. A quarter of patients diagnosed with MFS are new cases due to sporadic (de novo) mutation and do not have a family history.^7^

MFS is diagnosed using a clinical score called the systemic score and confirmed with genetic testing. The syndrome involves the cardiovascular system and is manifested by aortic aneurysms, aortic dissection, and mitral valve prolapse. It also affects the eye, causing dislocation of the ocular lens (ectopia lentis), manifested as myopia, and skeletal abnormalities due to the overgrowth of the long bones, such as tall stature and increased arm spam, scoliosis, kyphosis, pectus excavatum, pectus carinatum, hindfoot deformity, malar hypoplasia, and abnormal joint laxity, among others.^7^ Virtually every patient with MFS will have evidence of aortic disease at some point during their lifetime.10 Patients with MFS usually present with aneurysmal dilation of the aortic root and/or the ascending order, or type dissection. Women with MFS are at increased risk of dissection during pregnancy if the aortic diameter exceeds 4 centimeters.^9,10^

### Loeys-Dietz Syndrome

Loeys-Dietz syndrome is caused by pathogenic variants in the transforming growth factor receptor type 1 or 2, *TGFBR1* or *TGFBR2*. It is inherited in an autosomal dominant fashion. Other genes associated with this condition are *SMAD2, SMAD3, TGFB2*, and *TGFB3*.^11,12,13^

In LDS, there is arterial tortuosity of the head and neck vessels, aneurysms, hypertelorism, and bifid uvula.^14^ Other clinical features include cleft palate, translucent skin, malar hypoplasia, retrognathia, blue sclera, cervical spine abnormalities, joint laxity, and developmental delay. These patients also have pregnancy-related complications, including uterine rupture.^13^ The vascular disease in these patients is aggressive, with early mortality. Most patients have aortic root aneurysms (98%). In LDS, aortic dissection occurs at smaller diameters than in MFS (less than 5 centimeters).^13^ Therefore, surgical intervention is recommended at smaller diameters.2 Aneurysms also can develop in other vessels, including the subclavian, renal, superior mesenteric, hepatic, and coronary arteries.^13^

### Vascular Ehlers-Danlos Syndrome (type IV)

Vascular-type Ehlers-Danlos or Ehlers-Danlos type 4 is an autosomal dominant disorder caused by a defect in type 3 collagen encoded by the *COL3A1* gene.^15,16^ Clinical features include easy bruising, translucent skin, facial features (thin vermilion of the lips, micrognathia, narrow nose, and prominent eyes), and fragility of the arteries, uterus, and bowel. Most patients present with vascular dissection or rupture, gastrointestinal perforation, or organ rupture. The most common cause of death is arterial dissection or rupture primarily involving the thoracic or abdominal arteries, most frequently in the absence of aneurysms.^10,16^ Surgical repair is complicated by extreme tissue fragility.^17^ Pregnancy is associated with high risk of rupture of the uterus and arteries.

## Nonsyndromic Aortic Disease

Approximately 13% to 20% of patients with TAD but without MFS or LDS features have affected first-degree relatives.^2,5,6^ An inherited pattern for TAD has been identified in 21.5% of non-Marfan patients, with the predominant inheritance pattern being autosomal dominant (76.9%) with varying degrees of penetrance and expressivity.^18^ The age at presentation is lower compared to the mean age in sporadic cases, but not as young as in MFS (mean ages, 58.2 versus 65.7 versus 27.4 years, respectively).^5,18^

### Genetic Variants Associated with Nonsyndromic HTAD

Heterozygous pathogenic variants in single genes are responsible for HTAD in most families.^2,18,19^ Out of the 11 pathogenic genes for HTAD, the ones associated with the nonsyndromic HTAD are *ACTA2, MYH11, MYLK, LOX*, and *PRKG1*.^2,3,20^ *ACTA2*, the most frequently mutated gene causing HTAD, is associated with a high risk of presentation with acute aortic dissection.^21^ It has been reported that missense mutations in *ACTA2* are responsible for 14% of inherited ascending TAD and dissections. These mutations interfere with actin filament assembly and are predicted to decrease smooth muscle cells contraction, resulting in aortic tissues with medial degeneration, smooth muscle cell (SMC) hyperplasia and disarray, and stenotic arteries in the vasa vasorum due to medial SMC proliferation.^20^

## Guideline Recommendations for Genetic Testing

The 2022 ACC/AHA Aortic Disease Guideline recommends that patients with aortic root and/or ascending aortic aneurysms or aortic dissection and any of the following risk factors for HTAD be referred for genetic testing to identify pathogenic or likely pathogenic variants: syndromic features of MFS, LDS, vascular EDS, TAD presenting at age < 60 years, a family history of TAD or peripheral/intracranial aneurysms in a first or second degree relative, and/or a history of unexplained sudden death at a relatively young age in a first- or second-degree relative.^2^ Furthermore, in patients with an established pathogenic or likely pathogenic variant in a gene predisposing to HTAD, cascade genetic testing of at-risk relatives is recommended along with aortic imaging. The cascade testing of the offspring should be repeated as carriers of the pathogenic variant are identified. In patients who meet the clinical diagnostic criteria for MFS but do not have ectopia lentis, genetic testing is reasonable to exclude an alternative diagnosis of LDS.

Variants of unknown significance have not been confirmed to cause TAD and therefore should not be used to identify which family members are at risk or to guide clinical management.^2^ Genetic testing should always be accompanied by genetic counseling because results can be challenging to interpret, especially in variants of unclear significance and variable strength of association of specific genes with HTAD or pathogenic variants that behave differently, such as variants in the *FBN2* gene.^3^ In patients with syndromic and nonsyndromic HTAD who are contemplating pregnancy, genetic counseling is recommended.

### What Genes To Test?

A multigene panel comprising all genes known to cause HTAD is the recommended approach to testing. Testing panels include *FBN1, LOX, COL3A1, TGFBR1M, TGFBR2, SMAD3, TGFB2, ACTA2, MYH11, MYLK*, and *PRKG1*.^2,3^

## Conclusion

Once patients are diagnosed with HTAD, it is essential to identify and refer those at risk for heritable HTAD for genetic testing. Diagnosing associated genetic variants and syndromes is critical because the underlying genetics have an implication in medical management, surveillance, surgical intervention threshold, surgical risk, pregnancy risk, and risk of inheritance by the offspring. A panel that includes all genes known to cause HTAD is essential. Genetic counseling should always accompany genetic testing.

## Key Points

- Up 25% of patients with thoracic aortic diseases have an underlying Mendelian pathogenic variant.
- Identifying pathogenic genes is essential to identify family members at risk for the disease, guide surveillance and management, and evaluate associated vascular and systemic complications.
- Marfan syndrome, Loeys-Dietz syndrome, and vascular Ehlers-Danlos syndrome are the most recognized genetic syndromes associated with heritable thoracic aortic diseases (HTAD).
- *ACTA2* is the most frequently mutated gene causing nonsyndromic HTAD.
- Once an established pathogenic or likely pathogenic variant in a gene predisposing to HTAD is identified, cascade genetic testing of at-risk relatives is recommended.
